# THE FUTURE OF PARATRANSIT: A STUDY OF PROVIDER EXPERIENCES AND HOPES FOR CHANGE

**DOI:** 10.1093/geroni/igac059.706

**Published:** 2022-12-20

**Authors:** Shayna Gleason, Nina Silverstein

**Affiliations:** University of Massachusetts Boston, Boston, Massachusetts, United States; University of Massachusetts Boston, Boston, Massachusetts, United States

## Abstract

The present study examined paratransit managers’ perceptions of a changing transportation market, and what resources or supports they might need to adapt to evolving market expectations. As transportation network companies (e.g., Uber, Lyft) have emerged, the landscape of demand-responsive transportation has changed. However, the experiences of those closest to the operations of paratransit programs have been largely neglected in research. In-depth, semi-structured interviews were conducted with 16 managers of paratransit services. The resulting transcripts were coded iteratively using NVivo software, using both inductive and deductive approaches. We found that participants were already innovating and often wanted to be even more creative with their services, but were hampered by inadequate funding, driver shortages, regulatory or policy challenges, and other barriers. This study’s findings advance the literature toward greater understanding of how policymakers can leverage existing paratransit infrastructure to better serve the transportation needs of older adults and other transportation disadvantaged groups.

